# Genome-wide identification and comparative analysis of the *PYL* gene family in eight Rosaceae species and expression analysis of seeds germination in pear

**DOI:** 10.1186/s12864-022-08456-1

**Published:** 2022-03-25

**Authors:** Guoming Wang, Kaijie Qi, Xin Gao, Lei Guo, Peng Cao, Qionghou Li, Xin Qiao, Chao Gu, Shaoling Zhang

**Affiliations:** 1 Centre of Pear Engineering Technology Research, State Key Laboratory of Crop Genetics and Germplasm Enhancement, Nanjing Agricultural University, 210095 Nanjing, China; 2 Department of Cell Biology and Molecular Genetics, University of Maryland, College Park, MD USA

**Keywords:** *PYL*, ABA, Pear (*Pyrus bretschneideri*), Rosaceae, Seed germination, Abiotic stress

## Abstract

Abscisic acid (ABA) is a phytohormone that plays important roles in the regulation of plant growth, seed germination, and stress responses. The pyrabactin resistance 1-like (PYR/PYL) protein, an ABA receptor, was involved in the initial step in ABA signal transduction. However, the evolutionary history and characteristics of *PYL* genes expression remain unclear in Chinese white pear (*Pyrus bretschneideri*) or other Rosaceae species. In this study, 67 *PYL* genes were identified in eight Rosaceae species, and have been classified into three subgroups based on specific motifs and phylogenetic analysis. Intriguingly, we observed that whole-genome duplication (WGD) and dispersed duplication (DSD) have a major contribution to *PYL* family expansion. Purifying selection was the major force in *PYL* genes evolution. Expression analysis finds that *PYL* genes may function in multiple pear tissues. qRT-PCR validation of 11 *PbrPYL* genes indicates their roles in seed germination and abiotic stress responses. Our study provides a basis for further elucidation of the function of *PYL* genes and analysis of their expansion, evolution and expression patterns, which helps to understand the molecular mechanism of pear response to seed germination and seedling abiotic stress.

**Supplementary Information:**

The online version contains supplementary material available at 10.1186/s12864-022-08456-1.

## Introduction

Abscisic acid (ABA) plays a pivotal role in various aspects of plant growth and development, such as cell elongation and division, seed desiccation tolerance and dormancy, seed maturation and germination, root growth, leaf senescence, fruit ripening and adaptation to different biotic and abiotic stresses [1–4]. Moreover, ABA can crosstalk and interact with other phytohormones to regulate plant development and response to environmental cues [5, 6].

In plants, the ABA receptors Pyrabactin Resistance 1 (PYR1), PYR1-Like (PYL), and Regulatory Component of ABA Receptor (RCAR) (hereafter referred to as PYLs) function at the first step of the ABA signal pathway and activate downstream ABA signaling cascade [7, 8]. After binding ABA, PYLs subsequently interact with the clade A of protein phosphatase type 2Cs (PP2Cs). In the absence of ABA, PYL protein cannot bind to PP2C, thus preventing the activation of SUCROSE NONFERMENTING 1 (Snf1) - related protein kinase 2s (SnRK2s); whereas upon binding ABA, PYL can interact with PP2C and inhibits PP2C from dephosphorylating SnRK2 [9, 10]. In turn, phosphorylated SnRK2 activates downstream substrates, such as ABA-responsive element-binding factors (AREBs/ABFs) and ABA-responsive genes [11, 12].

As core regulators in ABA signaling, the function of *PYL* have been unraveled in many species, including *Arabidopsis thaliana* [7, 13], *Triticum aestivum* [14], *Oryza sativa* [15], *Zea mays* [16] and *Solanum lycopersicum* [17]. For instance, in *Arabidopsis*, a total of 14 PYL receptor proteins with the highly conserved START (star-related lipid transfer) domain have been identified and classified to 3 subfamilies [18, 19]. Different subfamilies play diverse roles in plant development and abiotic stresses, and functions of most *AtPYLs* have been verified comprehensively in *Arabidopsis*. *AtPYL5* and *AtPYL9* were identified to improve drought tolerance [13, 20]; *AtPYL5* increase photosynthesis rate in drought stress [21]; *AtPYR1*, *AtPYL2*, *AtPYL4* and *AtPYL5* are involved in regulating guard cell and stomatal closure in response to CO_2_ [7]; the *AtPYL8* and *AtPYL9* play a critical role in root growth and leaf senescence [13, 22].

*PYL* was also involved in seed germination, since ABA plays the primary role in seed dormancy and germination [8, 23]. Seed germination requires dynamic regulation of ABA signaling in a constantly changing environment. ABA prevents seed germination and post-germinative growth through the PYL receptors and PP2C co-receptors [24]. For example, *AtPYR1*/*PYL4* was involved in seed germination [24]; *AtPYL6* and *AtPYL13* have been verified to inhibit seed germination [25, 26]; *AtPYL11* and *AtPYL12* have been verified to control ABA-mediated seed germination [8]. In rice, the *OsPYL*/*RCAR5* was identified to be hypersensitive to ABA during seeds germination [27]. In addition, *PYL* as an ABA receptor, the expression of *PYLs* is precisely modulated at the transcriptional level. For instance, the expression of *PYLs* can be induced by multiple abiotic stresses, such as cold, drought and salinity [20, 28, 29]. However, the mechanism through which *PYLs* in plants are regulated at the transcriptional level remains unclear.

Although several *PYLs* have been identified in diverse plant species, the *PYL* family has not yet been systematically investigated in Rosaceae, which is an economically important family that includes many best-selling commercial fruit species, such as apple, pear, peach, strawberry and raspberry. Furthermore, PYL as an ABA receptor protein plays one of core role in ABA signaling. In this study, genome-wide identification and comparative analysis of the *PYL* family were performed in eight Rosaceae species. A total of 67 *PYL* genes were identified. The features, phylogenic relationships and evolution of *PYLs* were analyzed. qR-PCR of *PbrPYLs* was performed in pear seed under exogenous ABA treatment. In addition, the expression levels of *PbrPYL*s were further studied in pear seeding under heat, cold, drought, NaCl, or ABA treatment. Taking all together, this study provides a basis for revealing the roles of *PYLs* in seed germination and seedling development responding to stresses.

## Results

### Identification, phylogenetic relationship and features of *PYL* genes

To identify all the *PYL* gene members in eight Rosaceae species (Chinese white pear, European pear, Japanese apricot, apple, peach, strawberry, sweet cherry and black raspberry), the HMM (Hidden Markov Model) and BLASTp (e-value < = 1e-10) searches were carried out to search genome annotation using 14 *Arabidopsis thaliana PYL* [18] amino acid sequence as queries. The candidate *PYL* genes were validated by Pfam (http://pfam.xfam.org/) and Interproscan 63.0 (http://www.ebi.ac.uk/InterProScan/). The methods of screening and identification were referred to previous reports [19, 30]. After filtering out redundant and incomplete protein sequences, a total of 67 *PYL* genes were determined: 11 in Chinese white pear, 7 in European pear, 7 in Japanese apricot, 13 in apple, 7 in peach, 8 in strawberry, 6 in sweet cherry and 8 in black raspberry (Supplementary Table 1).

**Fig. 1:**
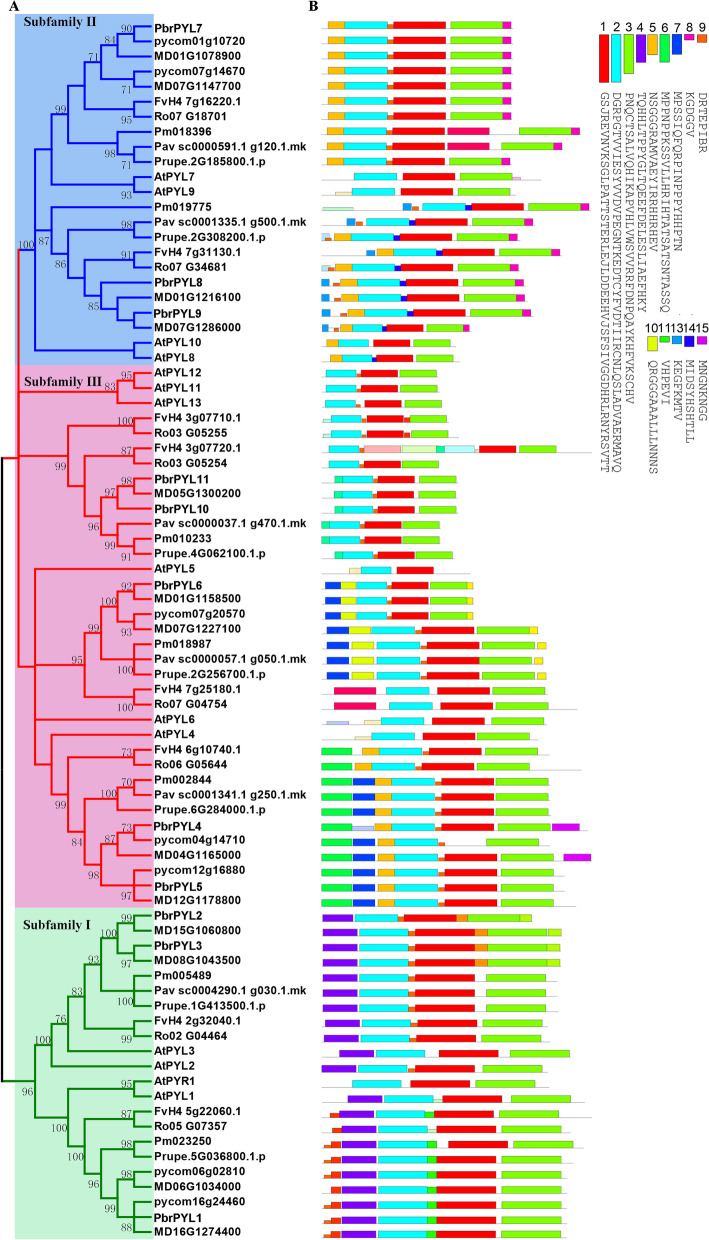
Phylogenetic relationship and conserved motifs of *PYL* genes. **A** phylogenetic relationship of *PYLs* was constructed in *Arabidopsis* and eight Rosaceae species. Tree was constructed by the Neighbor-Joining (NJ) algorithm. The green, blue, and red lines or boxes depict the subfamily I, II and III, respectively. **B** Distributions of conserved motifs in PYL proteins. Different color boxes indicated different motifs

To understand the phylogenetic relationship of the *PYL* proteins, the amino acid sequences encoded by *PYL* genes from eight Rosaceae species and *Arabidopsis* were used to construct a phylogenetic tree with the Neighbor-Joining (NJ) algorithm. Based on the classification of subfamilies in *Arabidopsis* [18], all the *PYL* genes in eight Rosaceae species were classified into three clades, designated Subfamily I, Subfamily II and Subfamily III (Fig. 1A). Overall, Subfamily I contained 22 members, Subfamily II contained 23 *PYLs*, and Subfamily III comprised 36 *PYLs* (Fig. 1A). *PYLs* genes grouping into the same subfamilies may perform similar functions. The *PYL* genes of Chinese white pear were named at *PbrPYL1-11* according to clustering relationship with *Arabidopsis* 14 *AtPYL* genes (*AtPYR1* and *AtPYL1-13*) in order. *PbrPYL2-11* were randomly distributed on 8 of 17 chromosomes and *PbrPYL1* on scaffold984.0 (Fig. 1A and Supplementary Table 1).

The MEME analysis was used to verify the classification of phylogenetic tree by analyzing the presence of conserved motifs in 67 *PYL* family members. 13 conserved motifs were detected from 67 PYL proteins, all of which contained motif 1, motif 2 and motif 3 (Fig. 1B). Remarkably, each of three subfamilies contained similar motifs; motif 4 was specific to subfamily I and motif 7 was only appeared in subfamily III, indicating that each subfamily is characterized by their respective conserved motifs, which may contribute to unique/specialized biological functions. All the *PbrPYL* genes include the PYR_PYL_RCAR domain (Supplementary Fig. S1). All of the members in subfamily II consists of two introns, whereas no introns were detected subfamilies I and III except *PbrPYL4* (Supplementary Fig. S1). Different *PYL* genes were grouped into each subfamily depicting similar motifs, domains and exon-intron structures, which confirmed their close evolutionary relationships and the classification of subfamilies. The detailed characteristics of the PYL proteins of *Arabidopsis* and eight Rosaceae species were listed in Supplementary Table 1. The molecular weights of 81 PYL proteins ranged from 17.57 kDa to 40.90 kDa. Protein isoelectric points (PI) ranged from pH 4.62 to pH 9.75 (Supplementary Table 1).

In addition, to better understand the potential function of the *PbrPYL* genes, we extracted promoter sequences of 2000 bp upstream from the initiation codons and identified cis-elements using the PlantCARE database. Some common cis-regulatory elements were briefly marked in promoter region, and the diversities of cis-elements related to the light, plant hormone and regulatory stress response suggested that expression may differ in response to development and stress (Supplementary Fig. S1).

### Synteny analysis of *PYL* gene family

Several gene duplication modes contribute to the formation of local gene clusters and evolution of protein-coding gene family, such as WGD, tandem and segmental [31]. Different modes of gene duplication were identified in eight Rosaceae species, and we detect duplicated *PYL* gene pairs to infer the evolutionary origins. All the *PYL* family members were assigned to five modes of gene duplication, including segmental duplication/ WGD, tandem duplication, proximal duplication, transposed duplication and dispersed duplication (Table 1 and Supplementary Table 2). A total of 87 duplicated gene pairs of *PYL* family members were identified in eight Rosaceae species, and the largest number is dispersed duplication (57 gene pairs, 65.5%), followed by WGD (26 gene pairs, 29.9%), and other duplications (4 gene pairs, 4.6%), suggesting that the modes of dispersed duplication and WGD mainly contribute to the expansion of the *PYL* gene family. Remarkably, pear and apple experienced the recent WGD event [32–34], so the number of *PYL* genes was more abundant in Chinese white pear and apple than in other species. The number of WGD duplications in apple and Chinese white pear were 11 and 6, but there were just one in Japanese apricot, European pear, black raspberry and strawberry. The number of dispersed duplication accounted for a large proportion of replication modes in each Rosaceae species, with the highest percentage in Japanese apricot and European pear (85.7%), and with the lowest percentage in apple (47.6%). Both WGD and dispersed duplication events play important roles in apple and Chinese white pear. The dispersed duplication events occurred more frequent than WGD in Rosaceae species except apple.

**Table 1:** Numbers of *PYL *gene pairs from different origins in 8 Rosaceae genomes

Species name	No. of PYL gene pairs	WGD	Tandem	Proximal	Transposed	Dispersed
*P. bretschneideri*	17	6 (35.3%)	0	0	1 (5.9%)	10 (58.8%)
*M. domestica*	21	11 (52.4%)	0	0	0	10 (47.6%)
*P. persica*	9	3 (33.3%)	0	0	0	6 (66.7%)
*P. mume*	7	1 (14.3%)	0	0	0	6 (85.7%)
*P. communis*	7	1 (14.3%)	0	0	0	6 (85.7%)
*R. occidentalis*	9	1 (11.1%)	1 (11%)	0	0	7 (77.8%)
*F. vesca*	10	1 (10%)	1 (10%)	0	1 (10%)	7 (70%)
*P. avium*	7	2 (28.6%)	0	0	0	5 (71.4%)

**Fig. 2:**
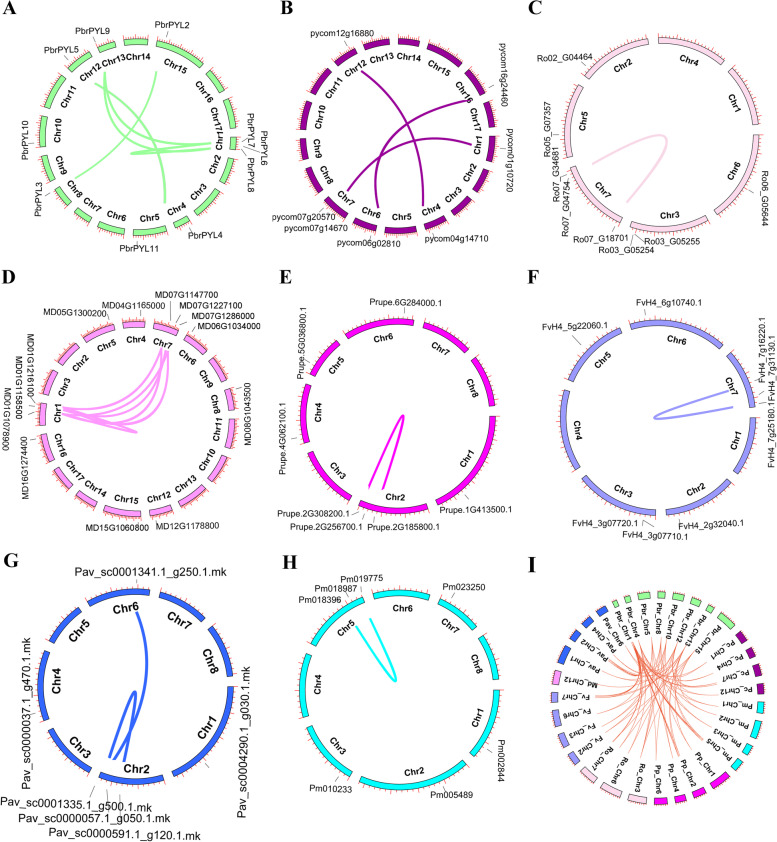
Chromosomal localization and syntenic relationships of *PYL* genes in eight Rosaceae species. **A** Chinese white pear; **B** European pear; **C** black raspberry; **D** apple; **E** peach; **F** strawberry; **G** sweet cherry; **H** Japanese apricot; **I**: The collinear correlation of the *PYL* is displayed between Chinese white pear and seven other Rosaceae species. *PYL* genes are mapped on different chromosomes and syntenic gene pairs are linked by colored lines

The *PYL* family genes were randomly distributed on different chromosomes in each species. For Chinese white pear, 10 of 11 *PbrPYL* genes were distributed on 8 of the 17 chromosomes while *PbrPYL1* was located on scaffold984.0 (Fig. 2A and Supplementary Table 1). Furthermore, intra-genomic collinearity of *PYL* gene family was investigated in each of eight Rosaceae species. 6 pairs was found in Chinese white pear, 3 pairs in European pear, 11 pairs in apple, 2 pairs in Japanese apricot, and one pair in strawberry, peach, sweet cherry and black raspberry (Fig. 2A-H and Supplementary Table 2). Further, the collinear correlation of the *PYL* genes between Chinese white pear and the other seven Rosaceae species were identified. A total of 81 pairs were found including 13, 1, 13, 13, 14, 12 and 14 collinear gene pairs between Chinese white pear with strawberry, apple, sweet cherry, Japanese apricot, peach, European pear and black raspberry (Fig. 2I and Supplementary Table 2). The results suggest that a conserved collinearity relationship between Chinese white pear and the other seven Rosaceae species.

### *PYL* genes evolved under strong purifying selection

Deleterious mutations can be eliminated by negative (purifying) selection and advantageous mutations can be accumulated by positive (Darwinian) selection [35]. To detect the selection pressure of *PYL* genes, we estimated the Ka, Ks and Ka/Ks values of paralogous *PYL* gene pairs in the eight Rosaceae species (Table 2). The strength and direction of selection pressure have been widely measured based on Ka/Ks ratio (Ka/Ks > 1: positive selection; Ka/Ks = 1: neutral evolution; Ka/Ks < 1: negative selection) [36]. All Ka/Ks ratios of the paralogous genes were less than one (Table 2), suggesting that purifying selection was the main driving force of *PYL* family gene evolution in Rosaceae species.

**Table 2:** Gene duplication events identified and Ka, Ks and Ka/Ks analysis in *PYL* gene family

Species names	Duplicated modes	Duplicate gene 1	Duplicate gene 2	Ka	Ks	Ka/Ks	*P*-Value
*Fragaria vesca*	WGD	FvH4_7g16220.1	FvH4_7g31130.1	0.111809	3.47965	0.0321323	2.44E-67
	TD	FvH4_3g07710.1	FvH4_3g07720.1	0.165144	1.18186	0.139732	9.47E-16
	TRD	FvH4_3g16470.1	FvH4_7g31130.1	0.0191826	1.0173994	0.0188545423	0.714904
	DSD	FvH4_2g32040.1	FvH4_5g22060.1	0.344001	3.50477	0.0981522	6.13E-29
		FvH4_3g07710.1	FvH4_7g25180.1	0.341964	3.3568	0.101872	0
		FvH4_3g07720.1	FvH4_6g10740.1	0.471614	3.64497	0.129388	0
		FvH4_5g22060.1	FvH4_6g10740.1	0.425737	3.4844	0.122184	0
		FvH4_6g10740.1	FvH4_7g25180.1	0.216804	3.4798	0.0623035	2.18E-56
		FvH4_7g16220.1	FvH4_7g25180.1	0.376761	3.50493	0.107495	0
		FvH4_7g31130.1	FvH4_6g10740.1	0.514944	3.70463	0.139	2.75E-35
*Malus domestica*	WGD	MD01G1078900	MD01G1216100	0.11464	3.5566	0.032233	4.33E-47
		MD01G1158500	MD07G1227100	0.0268639	0.314949	0.085296	2.40E-14
		MD01G1216100	MD07G1286000	0.0115856	0.133633	0.0866976	5.77E-08
		MD01G1078900	MD07G1147700	0.0116527	0.204397	0.0570102	5.83E-11
		MD01G1078900	MD07G1286000	0.109027	3.94655	0.0276258	1.02E-45
		MD01G1216100	MD07G1147700	0.106375	3.57085	0.0297899	4.11E-47
		MD04G1165000	MD12G1178800	0.0815507	0.404153	0.201782	9.88E-12
		MD06G1034000	MD16G1274400	0.0388825	0.40891	0.0950881	1.04E-14
		MD07G1147700	MD07G1286000	0.103343	2.90561	0.0355667	3.77E-45
		MD07G1227100	MD12G1178800	0.204621	3.1111	0.0657712	9.49E-30
		MD08G1043500	MD15G1060800	0.0463626	0.135679	0.341708	0.00562685
	DSD	MD01G1078900	MD07G1227100	0.379574	3.53939	0.107243	5.31E-39
		MD01G1158500	MD04G1165000	0.203633	3.56206	0.0571672	2.27E-37
		MD01G1216100	MD04G1165000	0.452985	3.6647	0.123608	3.47E-37
		MD04G1165000	MD07G1227100	0.190499	3.52175	0.0540923	1.83E-33
		MD05G1300200	MD07G1227100	0.34931	3.51504	0.0993757	5.12E-38
		MD06G1034000	MD08G1043500	0.350207	3.51566	0.0996134	0
		MD07G1147700	MD04G1165000	0.389925	3.52186	0.110716	2.95E-31
		MD08G1043500	MD16G1274400	0.33648	3.49283	0.0963343	0
		MD15G1060800	MD16G1274400	0.347433	3.541	0.0981174	8.11E-43
		MD16G1274400	MD07G1227100	0.416881	3.51859	0.11848	1.07E-40
*Prunus avium*	WGD	Pav_sc0000591.1_g120.1.mk	Pav_sc0001335.1_g500.1.mk	0.111293	3.47319	0.0320435	1.63E-53
		Pav_sc0000057.1_g050.1.mk	Pav_sc0001341.1_g250.1.mk	0.206769	3.45147	0.0599077	4.40E-42
	DSD	Pav_sc0000037.1_g470.1.mk	Pav_sc0001341.1_g250.1.mk	0.338981	3.35285	0.101102	3.39E-29
		Pav_sc0000057.1_g050.1.mk	Pav_sc0004290.1_g030.1.mk	0.427881	3.4789	0.122993	0
		Pav_sc0000591.1_g120.1.mk	Pav_sc0001341.1_g250.1.mk	0.384841	3.48093	0.110557	5.31E-29
		Pav_sc0001335.1_g500.1.mk	Pav_sc0001341.1_g250.1.mk	0.412724	3.52279	0.117158	0
		Pav_sc0001341.1_g250.1.mk	Pav_sc0004290.1_g030.1.mk	0.418773	3.45463	0.121221	1.32E-25
*Pyrus bretschneideri*	WGD	Pbr027457.1	Pbr013616.1	0.119033	3.57341	0.0333107	1.31E-44
		Pbr013616.1	Pbr010794.1	0.0157776	0.154226	0.102302	5.42E-08
		Pbr027457.1	Pbr010794.1	0.11267	3.57425	0.0315229	2.57E-52
		Pbr016128.1	Pbr000497.1	0.0796357	0.14881	0.535151	0.0896506
		Pbr028222.1	Pbr019415.1	0.0610577	0.46577	0.13109	1.79E-15
		Pbr019827.1	Pbr036422.1	0.0417345	0.113073	0.369095	0.0074364
	TRD	Pbr042468.1	Pbr036422.1	0.348505	3.50604	0.0994014	0
	DSD	Pbr000497.1	Pbr028222.1	0.422075	3.58091	0.117868	3.81E-26
		Pbr009570.1	Pbr019415.1	0.201551	3.53735	0.056978	4.32E-43
		Pbr010794.1	Pbr019415.1	0.522478	3.65444	0.142971	1.25E-36
		Pbr013616.1	Pbr019415.1	0.480095	3.65137	0.131483	5.60E-31
		Pbr016128.1	Pbr009570.1	0.345165	3.50627	0.0984424	8.68E-36
		Pbr019415.1	Pbr042468.1	0.385776	3.51598	0.109721	2.49E-37
		Pbr019827.1	Pbr042468.1	0.34476	3.50929	0.0982423	0
		Pbr027457.1	Pbr009570.1	0.376167	3.51597	0.106988	2.43E-37
		Pbr028222.1	Pbr042468.1	0.387427	3.52701	0.109846	2.84E-44
		Pbr036422.1	Pbr009570.1	0.392733	3.53164	0.111204	4.54E-24
*Pyrus communis*	WGD	pycom01g10720	pycom07g14670	0.0244846	0.16692	0.146685	9.06E-08
		pycom12g16880	pycom04g14710	0.0796933	0.362637	0.21976	3.32E-10
		pycom16g24460	pycom06g02810	0.0364341	0.429431	0.0848428	3.92E-15
	DSD	pycom01g10720	pycom07g20570	0.401604	3.53454	0.113622	6.03E-37
		pycom04g14710	pycom07g20570	0.236098	3.51749	0.0671212	5.83E-29
		pycom06g02810	pycom07g20570	0.427578	3.526	0.121264	2.32E-37
		pycom07g14670	pycom07g20570	0.430594	3.50067	0.123003	1.81E-36
		pycom07g20570	pycom12g16880	0.217137	3.00525	0.0722526	6.48E-30
		pycom12g16880	pycom16g24460	0.400614	3.5472	0.112938	2.42E-38
*Prunus mume*	WGD	Pm018396	Pm019775	0.111453	3.47718	0.0320528	1.07E-55
	DSD	Pm002844	Pm018987	0.211125	3.46082	0.0610044	1.70E-38
		Pm005489	Pm023250	0.337261	3.5912	0.0939129	3.91E-44
		Pm010233	Pm018987	0.303213	3.28914	0.092186	0
		Pm018396	Pm018987	0.366869	3.45993	0.106034	0
		Pm018987	Pm023250	0.423619	3.43084	0.123474	0
		Pm019775	Pm018987	0.42043	3.61373	0.116342	1.06E-42
*Prunus persica*	WGD	Prupe.2G185800.1	Prupe.2G308200.1	0.110113	3.58704	0.0306975	2.88E-52
	DSD	Prupe.1G413500.1	Prupe.5G036800.1	0.350907	3.56861	0.0983314	2.98E-41
		Prupe.2G185800.1	Prupe.2G256700.1	0.371103	3.44492	0.107725	0
		Prupe.2G256700.1	Prupe.6G284000.1	0.213853	3.4239	0.062459	1.50E-40
		Prupe.2G308200.1	Prupe.6G284000.1	0.401603	3.54339	0.113339	0
		Prupe.4G062100.1	Prupe.6G284000.1	0.389699	3.47765	0.112058	1.04E-22
		Prupe.5G036800.1	Prupe.2G256700.1	0.42	3.4189	0.122847	0
*Rubus occidentalis*	WGD	Ro07_G18701	Ro07_G34681	0.101014	3.49716	0.0288844	2.92E-50
	TD	Ro03_G05255	Ro03_G05254	0.121703	1.65984	0.0733222	3.83E-22
	DSD	Ro02_G04464	Ro05_G07357	0.355936	3.46687	0.102668	8.77E-32
		Ro03_G05254	Ro07_G04754	0.302499	3.42801	0.0882432	1.57E-41
		Ro03_G05255	Ro07_G04754	0.33339	3.44744	0.0967064	2.86E-36
		Ro05_G07357	Ro06_G05644	0.399903	3.4264	0.116712	4.02E-28
		Ro06_G05644	Ro07_G04754	0.31105	3.54625	0.0877124	9.65E-27
		Ro07_G04754	Ro07_G18701	0.356946	3.48012	0.102567	4.36E-41
		Ro07_G34681	Ro07_G04754	0.439223	3.51407	0.12499	0

### Expression pattern of *PYL* family genes in different pear tissues

**Fig. 3:**
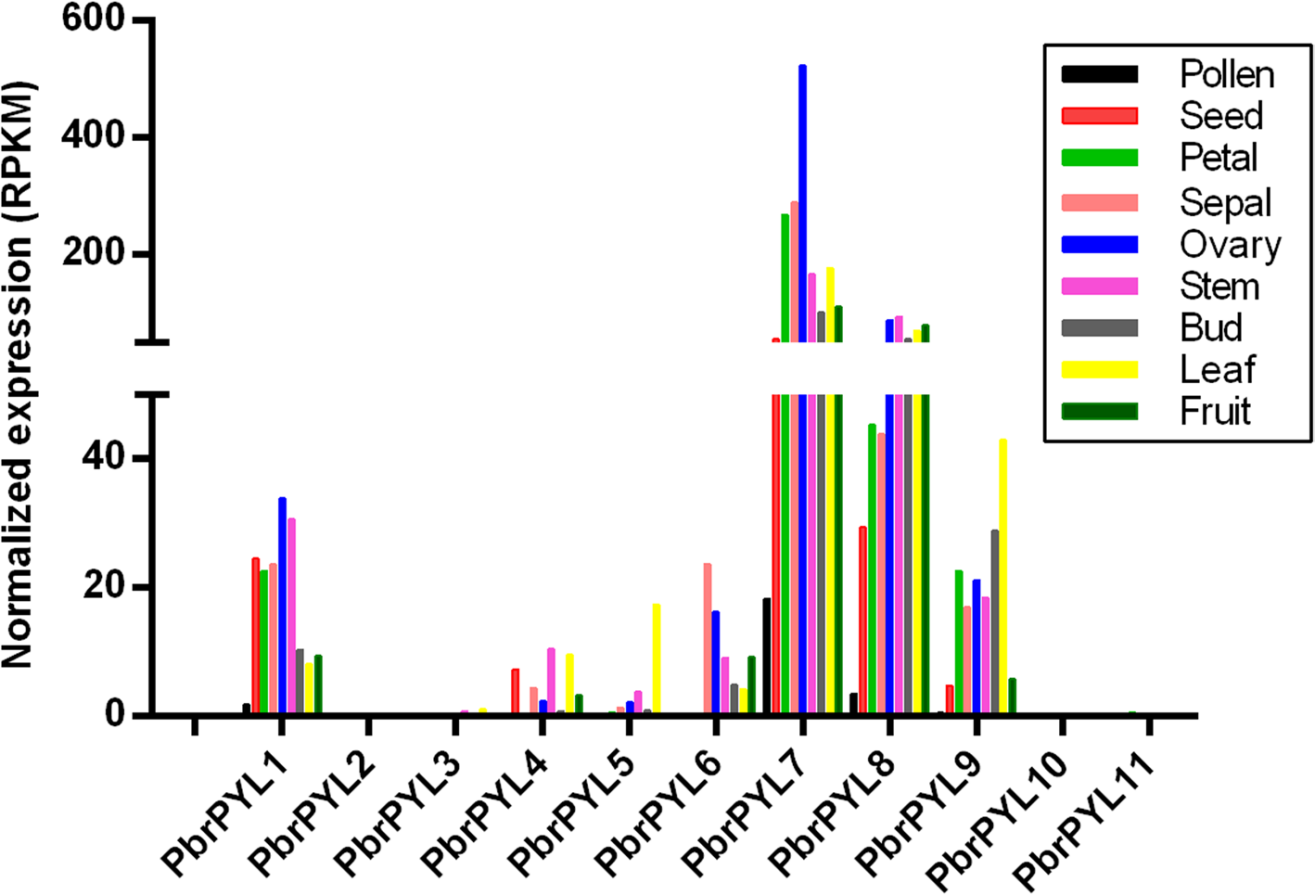
Relative gene expression of *PbrPYLs* in pollen, seed, petal, sepal, overy, stem, bud, mature leaves, and mature fruit was determined by RNA-Seq from Dangshan pear. RPKM, reads per kilobase of exon model per million mapped reads

To analyze the expression levels of *PYL* family genes in different pear tissues, previously published RNA-seq data was analyzed including mature pollen, seed, sepal, petal, ovary, bud, stem, leaf and fruit (Fig. 3 and Supplementary Table 3). The transcript abundances of four *PYL* genes (*PbrPYL2*, *PbrPYL3*, *PbrPYL10* and *PbrPYL11*) in all experimental tissues were hardly detected. *PbrPYL1*, *PbrPYL7*, *PbrPYL8* and *PbrPYL9* were expressed in all the different pear tissues, whereas *PbrPYL7* and *PbrPYL8* were expressed at higher levels, indicating their roles in different tissue growth and development. Most *PbrPYL* genes were expressed in mature fruits except *PbrPYL2*, *PbrPYL3*, *PbrPYL10* and *PbrPYL11.* Most *PbrPYL* genes presented no or very low expression levels in pollen except *PbrPYL7, PbrPYL5* and *PbrPYL9* showed higher expression levels in leaf than other tissues. Moreover, *PbrPYL1*, *PbrPYL4*, *PbrPYL7*, *PbrPYL8* and *PbrPYL9* were expressed in ungerminated mature seeds.

### Expression analysis of *PbrPYLs* during ABA treatment and seed germination

**Fig. 4:**
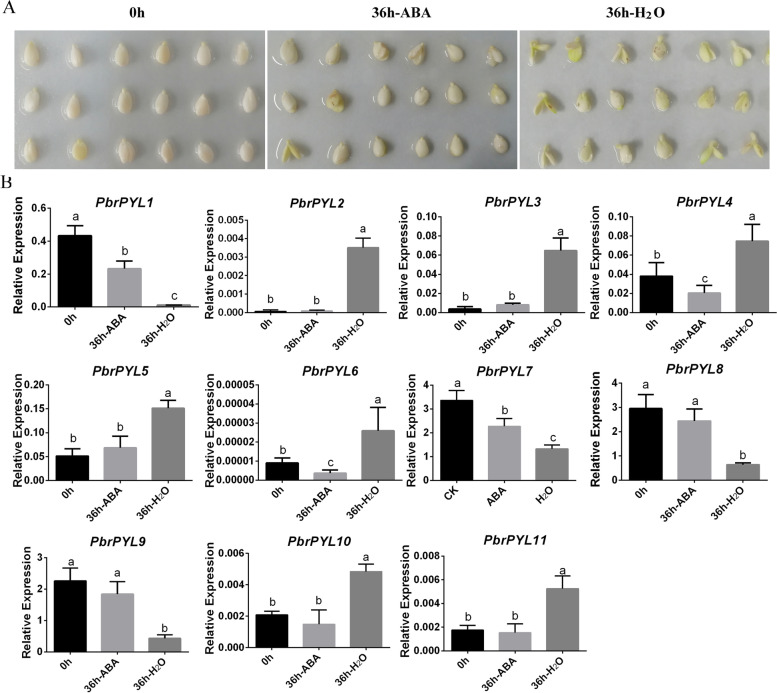
Seed treatment and expression analysis of *PbrPYL* genes. **A** Coat peeled from seed and treated with ABA and H2O. **B** Expression levels of 11 *PbrPYL* genes under 0 h (dormant embryos), 36 h ABA (dormant embryos) and 36 h H2O (germinated embryos)

Previous research has shown that dormant seed coats produce ABA to suppress embryo germination [37, 38]. Therefore, pear seed coats were peeled to prevent ABA interference and accelerate germination. As expected, the uncoated seeds treated with water successfully germinated in 36 h (Fig. 4A). However, the seeds treated with ABA for 36 h did not germinate as well as control treated for 0 h (Fig. 4A). As the ABA receptors, the *PYL* family has been reported to play an important role in seed germination [18, 39]. To better reveal the functions of *PYLs* in the ABA signal pathway during the seed germination, 11 *PbrPYL* genes were detected using qRT-PCR. Consistent with the transcriptome data in seed (Fig. 3), just *PbrPYL7*, *PbrPYL8* and *PbrPYL9* genes of the 11 *PYL* genes were much greater expressed than those of the other *PYLs* expressed in seeds (Fig. 4B). Moreover, *PbrPYL7*, *PbrPYL8* and *PbrPYL9* of subfamily II were significantly lower expressed in the germinated embryos (36 h-H_2_O) than in the dormant embryos (0 and 36 h-ABA treatment) (Fig. 4B). Conversely, the expression of other *PbrPYL* genes except the *PbrPYL7*, *PbrPYL8* and *PbrPYL9* genes were low or and almost not expressed. Although the low expression of *PbrPYL2*, *PbrPYL3*, *PbrPYL4*, *PbrPYL5*, *PbrPYL6*, *PbrPYL10* and *PbrPYL11*, they were most strongly expressed in the germinated embryos than dormant embryos (Fig. 4B). The expression of *PbrPYL2*, *PbrPYL3*, *PbrPYL4*, *PbrPYL5*, *PbrPYL6*, *PbrPYL10* and *PbrPYL11* increased dramatically at 36 h after germination, whereas that of *PbrPYL1*, *PbrPYL7*, *PbrPYL8* and *PbrPYL9* decreased substantially. Upon exogenous ABA treatment during germination, the trends of gene expression were somewhat changed (Fig. 4B). Therefore, ABA can inhibit germination of the uncoated seed. Meanwhile, this significant change of *PbrPYL* genes indicates that *PYLs* play critical and various roles in ABA-mediated seed germination.

### Expression analysis of *PbrPYLs* in pear seedling under different treatments

**Fig. 5:**
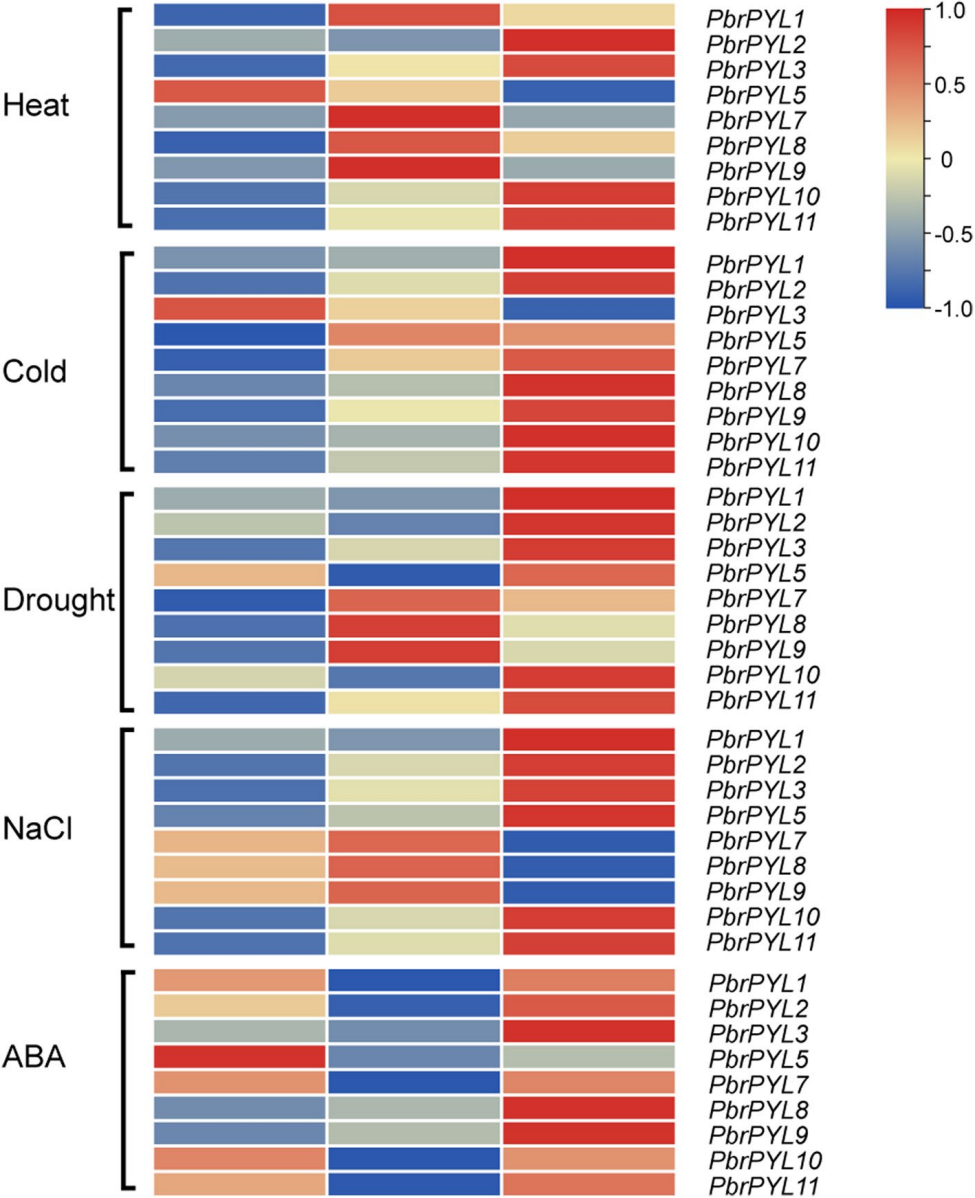
Expression analysis of *PbrPYL* genes under heat, cold, drought, NaCl, and ABA treatments in pear seedling. The bars display the relative gene expression levels, calculated based on the 2^−ΔΔ^Ct method. The expression levels are equal to the mean values and transform log_2_ values

In this study, the expression patterns of 11 *PbrPYL* family genes were detected by qRT-PCR, and the results showed transcriptional changed under heat, cold, drought, NaCl, or ABA treatment, suggested that the response of *PbrPYLs* to multiple stresses is a dynamic process (Fig. 5 and Supplementary Table 5). The expression levels of *PbrPYL7, PbrPYL8* and *PbrPYL9* remain very high under different treatments, while *PbrPYL4* and *PbrPYL6* were hardly detected during all treatments. Although other six *PbrPYL* genes were expressed at lower levels, their expression levels were altered at different time points in response to various treatments (Fig. 5). For heat treatment, three genes of *PbrPYL7*, *PbrPYL8* and *PbrPYL9* tended to be observably up-regulated at 12 h and decreased at 24 h. Four genes (*PbrPYL2*, *PbrPYL3*, *PbrPYL10* and *PbrPYL11*) were significantly up-regulated at 24 h. *PbrPYL5* was induced rapidly at 6 h and decreased at 12 and 24 h (Fig. 5). Under cold treatment, the genes of *PbrPYL1*, *PbrPYL2*, *PbrPYL7*, *PbrPYL8*, *PbrPYL9*, *PbrPYL10* and *PbrPYL11* did not change significantly at 6 h after treatment, but up-regulated at 24 h (Fig. 5). Under drought or NaCl treatment, most genes had similar expression patterns, *PbrPYL7*, *PbrPYL8* and *PbrPYL9* genes were significantly up-regulated at 12 h, while there was no significant difference at 24 h. Other genes were significantly up-regulated at 24 h (Fig. 5). Under ABA treatment, the genes of *PbrPYL1*, *PbrPYL2*, *PbrPYL5*, *PbrPYL7*, *PbrPYL10* and *PbrPYL11* were up-regulated rapidly at 6 h. All the genes except *PbrPYL5* were significantly up-regulated at 24 h (Fig. 5). In addition, three highly expressed genes, *PbrPYL7*, *PbrPYL8* and *PbrPYL9*, had similar expression trend at 12 h under heat, drought and NaCl treatments, and observably up-regulated at 24 h under cold and ABA treatments. Taken together, all the nine genes were induced by different stresses, but the diverse expression pattern of *PbrPYL* genes may suggest that these genes may be critical to abiotic and hormone stress responses.

### Subcellular localization of PbrPYL proteins

**Fig. 6:**
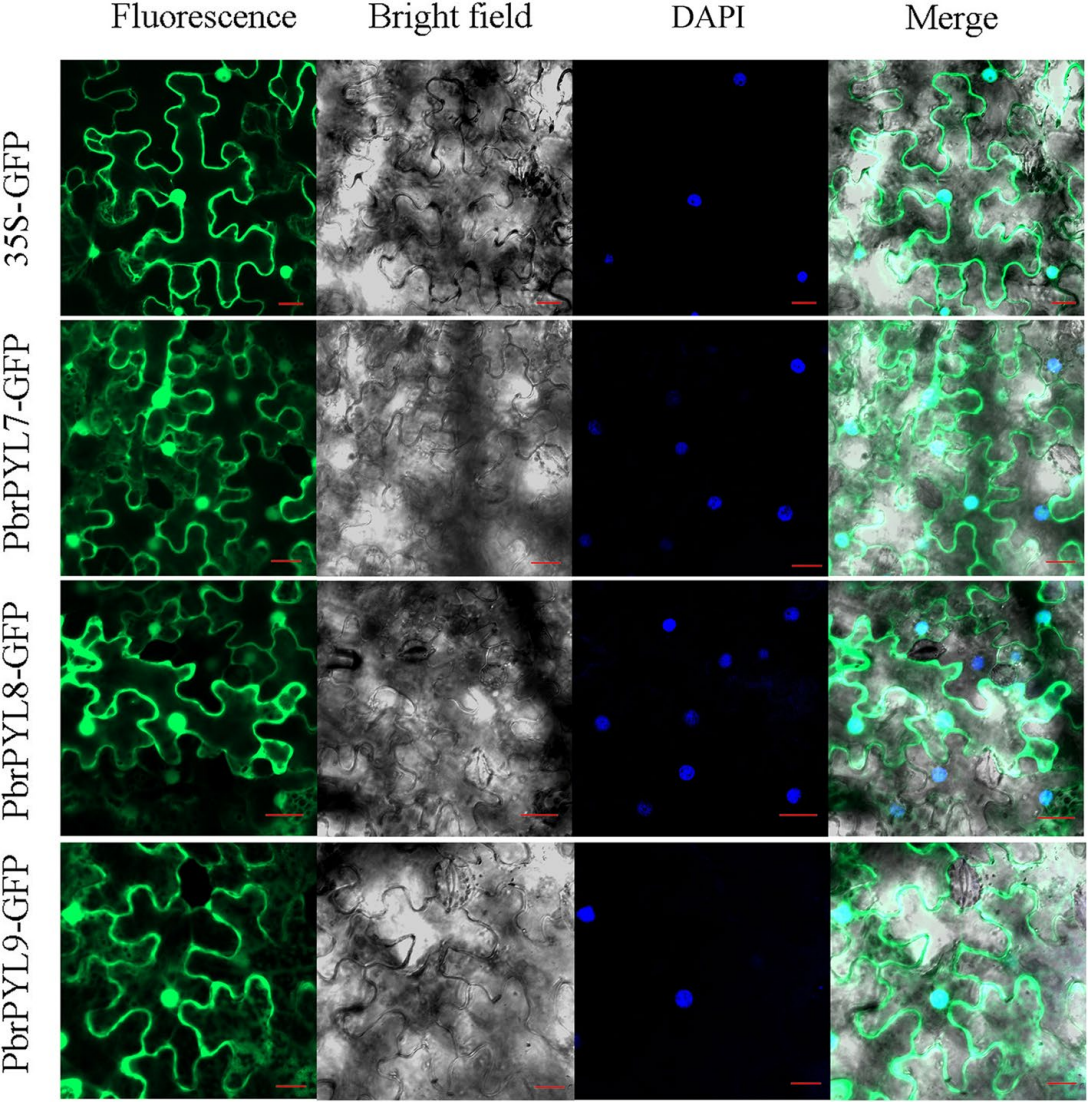
Subcellular localization of the fusion protein PbrPYLs-GFP in *N. benthamiana* leaves. The vector 35 S-GFP was used as the control. Bar = 20 μm

To investigate the subcellular localization of the PbrPYL proteins, three highly expressed genes *PbrPYL7/8/9* were cloned and individually constructed into a recombinant plasmid. Microscopic visualization showed that the GFP fluorescence signal of the positive control 35 S-GFP was distributed the whole cell, whereas the fluorescence of PbrPYL7/8/9-GFP was detected in the cytosol and the nucleus (Fig. 6). The results showed that PbrPYL proteins were localized in both the cytoplasm and the nucleus, which is consistent with the previous research [20, 27]. PP2C protein is at the downstream of PYL protein and interacts with PYL, which is located in the nucleus [27]. PP2C complexes were also localized to the nucleus, even if the interacting partners PYL protein were not exclusively localized in the nucleus [20, 27]. This indicates that the PYL may function as a nuclear ABA receptor in the regulation ABA-dependent gene expression.

## Discussion

### Gene structure and evolution

ABA is directly perceived by the PYL receptor, which plays an important role in initiating the ABA signaling pathway [18, 40]. The identification and function of *PYL* genes have been studied in many model species, such as *Arabidopsis* [7], *Oryza sativa* [15, 27] and *Solanum lycopersicum* [41]. However, the knowledge of the *PYL* gene family is very limited in the Rosaceae species. In present study, a genome wide comprehensive analysis of *PYL* genes from 8 Rosaceae species was carried out, and their potential role in pear seed development was subsequently investigated. A total of 67 *PYLs* in the 8 Rosaceae species were identified based on the similarity to 14 *AtPYLs*. The numbers of *PYLs* across 8 different Rosaceae species ranges from 6 to 13, including 13 in apple, 11 in Chinese white pear, 8 in strawberry, 8 in black raspberry, 7 in European pear, 7 in Japanese apricot, 7 in peach and 6 in sweet cherry. Meanwhile, chromosomal distribution of *PYL* genes across 8 Rosaceae species showed uneven distribution (Fig. 2). Based on phylogenetic analysis with *Arabidopsis* homologs, 67 *PYLs* can be broadly classified into three clusters: subfamilies I, II and III (Fig. 1A), which is consistent with the classification of *PYL* genes in other plant species [28, 29]. The number and composition of conserved motifs varied in each *PYL* subfamilies (Fig. 1B). All 67 PYL protein contained motif 1, motif 2 and motif 3, indicating a highly conserved function of this family. Phylogeny analysis of *PYL* genes showed that similar motifs exist in each subfamily, such as motif 4 and motif 7 were specific to subfamily I and subfamily III, respectively (Fig. 1). *PYL* genes of the same subfamily have similar unique/specialized biological functions, but their functions remain unclear.

Gene duplication events can be generally divided into five types, including WGD, TD, PD, DSD and TRD, which could drive gene family expansion in eukaryotes [42, 43]. WGD events can generate a large number of duplicate genes in a short period of time [44]. WGD and small-scale duplication events are not only the main features of eukaryotic genome evolution, but also the main driving force of new functions in the genome and genetic evolutionary system [43]. Gene families, such as heat-shock transcription factors, *SWEET* and *F-box* families, expanded primarily through WGD and DSD [45–47]. However, the expansions of *WRKY* and *AP2/ERF* gene families mainly resulted from TD events [48, 49]. In this study, WGD and DSD were found to be the main driving forces for the expansion of the *PYL* gene family in 8 Rosaceae species. Meanwhile, the number of DSDs account for a large proportion of gene duplications and made primary contributions in the expansion of *PYL* genes (Table 1). In addition, evolutionary analysis based on the Ka, Ks and Ka/Ks, suggesting that purifying selection were the primary evolutionary force imposing on *PYL* family genes in 8 Rosaceae species (Table 2).

### Regulation of gene expression

Analyzing the expression pattern of pear *PYL* genes across the broad spectrum of various tissues, developmental stages and stress treatments will help further understand the physiological and developmental functions of *PbrPYL*. The *PYL* genes expression pattern in different tissues has been studied in many species. The expression levels of most *PYL* genes in soybean seeds were higher than those in other tissues [50]. The transcription level of *PYLs* showed much higher in seeds of oilseeds during germination than in other various tissues [51]. The transcription level of *PYLs* was highly abundant in latex of rubber tree [52]. *PYLs* were expressed at a higher level in the callus of *B. rapa* than in other tissues [53]. We analyzed the *PbrPYL* genes expression patterns in the various tissues of ‘Dangshang’ pear. The result shows that *PbrPYL1, PbrPYL4, PbrPYL5, PbrPYL6, PbrPYL7, PbrPYL8* and *PbrPYL9* showed higher expression potential in different tissues, suggesting that these *PYLs* have multiple roles throughout the growth and development. Furthermore, we found that all 3 members (*PbrPYL7*, *PbrPYL8* and *PbrPYL9*) of subfamily II showed much higher expression potential in different tissues (Figs. 3 and 4). However, other *PbrPYL* genes in all of the various tissues were nearly zero, indicating that the functions of these *PbrPYL* genes are not required (Fig. 3). Meanwhile, the results showed that only *PbrPYL1, PbrPYL4, PbrPYL7, PbrPYL8* and *PbrPYL9* were highly expressed in dormant embryos (Fig. 3).

ABA acts as a primary mediator in seed dormancy and germination [37, 38]. In this study, uncoated seeds germinated for 36 h under water treatment, but there was no significant change at the same time under ABA treatment (Fig. 4A). This indicates that ABA inhibited seeds germination, which was consistent with previous studies [24, 38]. *PYL* gene family as ABA receptors starts from ABA sensing and plays an important role in ABA-mediated seed germination. The *OsPYL7*, *OsPYL8* and *OsPYL9* in rice have been shown to play important role during seed germination and development stage [19]. *OsPYL/RCAR5* and *PYL8/RCAR3* may perform critical biological functions in seed germination and seedling growth in rice and *Arabidopsis* [27, 54]. *PYL11* and *PYL12* were expressed specifically in mature seeds, which positively modulate ABA-mediated seed germination in *Arabidopsis* [8]. The *Arabidopsis pyr1*, *pyl1*, *pyl2* and *pyl4* single mutants were not sensitive to ABA during seed germination, while they quadruple mutant was strongly insensitive to ABA [18]. *PYL13* overexpression mutants were sensitive to ABA during seed germination [39]. *PYR1*, *PYL1*, *PYL2* or *PYL4* overexpression mutants can increase sensitivity to ABA-mediated inhibition during seed germination [55]. Among the 11 *PYL* genes detected in pear, the expression of 7 *PbrPYL* were up-regulated during seed germination and 4 *PbrPYL* were down-regulated by exogenous ABA. This result indicates the diversity of the expression patterns and multiple roles of *PYLs* in ABA signaling.

ABA has been reported to play crucial roles in responding to multiple abiotic stresses, such as heat, cold, drought and salinity [56, 57]. ABA was produced rapidly in response to multiple stresses and then regulates the stress response. PYL as the ABA receptor was involved in the initial step under multiple abiotic stresses [20, 28, 29]. In this study, qRT-PCR analysis showed that all the *PbrPYLs* were almost up-regulated by one or more different abiotic stresses (heat, cold, drought and NaCl) and ABA treatment at seedling stage, except *PbrPYL4* and *PbrPYL6*, the expression of which were too low to be detected. This suggests that *PYLs* play important roles in stress responses and ABA treatment of pear. By contrast, the expression patterns of all 3 subfamily II members (*PbrPYL7*, *PbrPYL8* and *PbrPYL9*) were up-regulated many folds than control under four different abiotic stresses and ABA treatment. These *PbrPYLs* may be utilized to improve the tolerance of pear seedling under abiotic stresses and ABA treatment.

## Conclusions

In conclusion, 67 *PYL* genes were identified in eight Rosaceae species and classified into three subgroups. WGD and DSD were major contributions to PYL family expansion. Purifying selection was the major force in *PYL* gene evolution. The qRT-PCR analyses of 11 *PbrPYL* genes revealed multifaceted critical roles of *PYL* in seed germination as well as abiotic stress responses. This study provides a basis for further elucidation of the function of *PYL* genes and analysis of their expansion, evolution and expression patterns, which helps to understand the molecular mechanism of pear in response to seed germination and seedling abiotic stress.

## Materials and methods

### Identification of *PYL* genes in pear and other Rosaceae species

To identify the *PYL* gene family in Chinese white pear and other seven Rosaceae species, multiple database searches were performed. The Chinese white pear (*Pyrus bretschneideri*) genome sequence was retrieved from the Pear Genome Project (http://peargenome.njau.edu.cn/) [34]. The Japanese apricot (*Prunus mume*) genome sequence was obtained from *Prunus mume* Genome Project (http://prunusmumegenome.bjfu.edu.cn/index.jsp). The apple (*Malus domestica*), the European pear (*Pyrus communis*), peach (*Prunus persica*), strawberry (*Fragaria vesca*), sweet cherry (*Prunus avium*) and black raspberry (*Rubus occidentalis*) genomic datasets were downloaded from the Genome Database of Rosaceae (GDR) (http://www.rosaceae.org). *Arabidopsis PYL* genes were downloaded from TAIR (http://www.arabidopsis.org/) [18] and used as query to identify *PYL* members in eight Rosaceae species (Supplementary Table 1). A local TBLASTN (version 2.2.26, Bethesda, MD, USA) search was performed and the E-value threshold was set at 1 × e^− 10^ to obtain the candidate *PYL* genes.

### Phylogenic and conservative motif analysis of *PYL* family members

The phylogenetic trees were constructed by MEGA7.0 [58] using the full-length protein sequences of PYL from eight Rosaceae species. Neighbor-Joining (NJ) algorithm with a matrix of pairwise distances estimated was performed for amino acid sequences. 1000 replicates were carried out for Bootstrap analysis. All full-length amino acid sequences of the PYLs were analyzed for conserved motifs by online MEME (http://meme-suite.org/). The conserved domains of PYL were analyzed by online Conserved Domain Database (CDD) (https://www.ncbi.nlm.nih.gov/cdd). The gene structure of *PbrPYLs* was analyzed online by the GENE Structure display (GSDS 2.0) website (http://gsds.gao-lab.org/). The 2000 bp genomic sequences upstream of *PbrYPLs* were extracted, and the cis-acting elements in the promoter region were analyzed by PlantCARE (http://bioinformatics.psb.ugent.be/webtools/plantcare/html/).

### Chromosomal location and synteny analysis of *PYL* genes

Chromosomal location information of *PYL* genes were obtained from genome annotation files of eight Rosaceae species and displayed using the TB-tools software [59]. The synteny analysis among eight Rosaceae genomes was performed using the method implemented in the PGDD (http://chibba.agtec.uga.edu/duplication/) [60]. Potential homologous gene pairs were identified using BLASTP (E < 1 e–5, top 5 matches). And the homolog pairs and gene location information were analyzed by MCScanX to identify syntenic chains [61]. Afterward, DupGen_finder was further employed for whole-genome (WGD), tandem (TD), proximal (PD), transposed (TRD) and dispersed (DSD) duplications of *PYL* family genes [62]. The results were visualized using the TB-tools software [59].

### Calculating values of Ka, Ks and Ka/Ks

The nonsynonymous (Ka) and synonymous (Ks) substitution rates and Ka/Ks ratios were calculated for valid gene pairs by KaKs_Calculator 2.0 with a model-averaged method and default parameters [63].

### Expression analysis of *PYL* in different tissues

The transcriptome data of ‘Dangshansuli’ different tissues were used to analyze the expression patterns of *PYL* family members. The transcriptome data of different tissues were obtained from our previously published studies and unpublished data [34, 64, 65], including pollen, seed, petal, sepal, ovary, stem, bud, leaf and fruit. RNA-Seq raw data were obtained from the Sequence Read Archive (https://www.ncbi.nlm.nih.gov/bioproject/) with the accession numbers PRJNA503323 and PRJNA498777 [64]. The RPKM values were used to estimate the gene expression abundances.

### Plant materials treatment and qRT-PCR analysis

‘Cuiguan’ pear (*Pyrus pyrifolia* Nakai) seeds were obtained from the pear germplasm orchard of Pear Engineering Technology Research Center of Nanjing Agricultural University situated at Baima in Nanjing with the permission. Seeds with seed coat peeled were treated with water or 1 ppm ABA. Seeds were placed on moistened gauze in a growth chamber at 25 ± 1 °C with dark and 60% relative humidity, and then collected in 0 h, 36 h (ABA treatment) and 36 h (water treatment). Germinated seeds were sowed in plastic pots. Seedlings were grown in a growth chamber for five weeks (the photoperiod 16/8 h, the temperature 25 ± 1 °C) and then exposed to various stresses. Seedlings were exposed to 4 and 37 °C for cold and heat treatment, respectively. For NaCl, drought stress and ABA treatments, the seedlings were cultured in 200 mM NaCl, 20% PEG 6000, and 100 µM ABA. The conditions of stress treatments were referenced by previous reports [19]. All treated samples and blank controls were collected in continuous time intervals of 6, 12 and 24 h, respectively. The samples were immediately frozen in liquid nitrogen, and stored at − 80 °C until use. Total RNA was extracted using RNAprep Pure Plant Kit (Tiangen, Beijing, China). The extracted total RNAs were synthesized the first-strand cDNA using TransScript One-Step gDNA Removal and cDNA synthesis Supermix (TransGen, Beijing, China). The primers of all the *PYL* family genes were designed using Primer Premier 6.0 and listed in Supplementary Table 4. The qRT-PCR was carried out using a LightCycler 480 SYBRGREEN I Master (Roche, USA). The tubulin gene of pear was used as the reference. All reactions were carried out with three independent biological replicates. The genes expression levels were calculated with the 2^−ΔΔCt^ method.

### Subcellular localization of the *PYL* genes

The full-length *PbrPYL* coding sequences without the termination codon were cloned from pear, and directionally inserted into the pCAMBIA1300-35 S: CDS-GFP vector. Primers used for cloning were listed in Supplementary Table 4. The recombinant plasmids and the control plasmid (pCAMBIA1300-35 S alone) were individually transformed into *Agrobacterium tumefaciens* strain GV3101. 30-day-old tobacco (*Nicotiana benthamiana*) leaves were used for agrobacterial injection according to the published protocol [66]. DAPI staining was used to indicate the nucleus. The fluorescence was imaged using a confocal microscope LSM780 (Zeiss LSM 780, Germany).

## Supplementary Information

**Additional file 1.**

**Additional file 2.**

**Additional file 3.**

**Additional file 4.**

**Additional file 5.**

**Additional file 6.**

## Data Availability

All relevant data analyzed during this study are included in this article and in Additional files. RNA-Seq raw data were obtained from the Sequence Read Archive (https://www.ncbi.nlm.nih.gov/bioproject/) with the accession numbers PRJNA503323 and PRJNA498777.

## Abbreviations

ABA: Abscisic acid
AREBs/ABFs: ABA-responsive element-binding factors
DSD: dispersed duplication
GDR: Genome database for Rosaceae
Ka: nonsynonymous substitution rates
Ks: synonymous substitution rates
MEME: Multiple EM for Motif Elicitation
ML: Maximum likelihood
NJ: Neighbor-joining
PP2C: protein phosphatase type 2 C
PD: proximal duplication
PGDD: Plant genome duplication database
PYR/PYL: pyrabactin resistance 1-like
RPKM: reads per kilobase of exon model per million mapped read
SnRK2: SUCROSE NONFERMENTING 1 (Snf1) - related protein kinase 2
TD: tandem duplication
TRD: transposed duplication
WGD: Whole-genome duplication
