# Supplementary material for: Genome-wide identification and comparative analysis of the PYL gene family in eight Rosaceae species and expression analysis of seeds germination in pear
Source: BMC Genomics. 2022 Mar 25;23:233. doi: 10.1186/s12864-022-08456-1 (PMC8957196; doi:10.1186/s12864-022-08456-1)
Supplement: Supplementary file 1 — Additional file 1. [file 12864_2022_8456_MOESM1_ESM.docx]

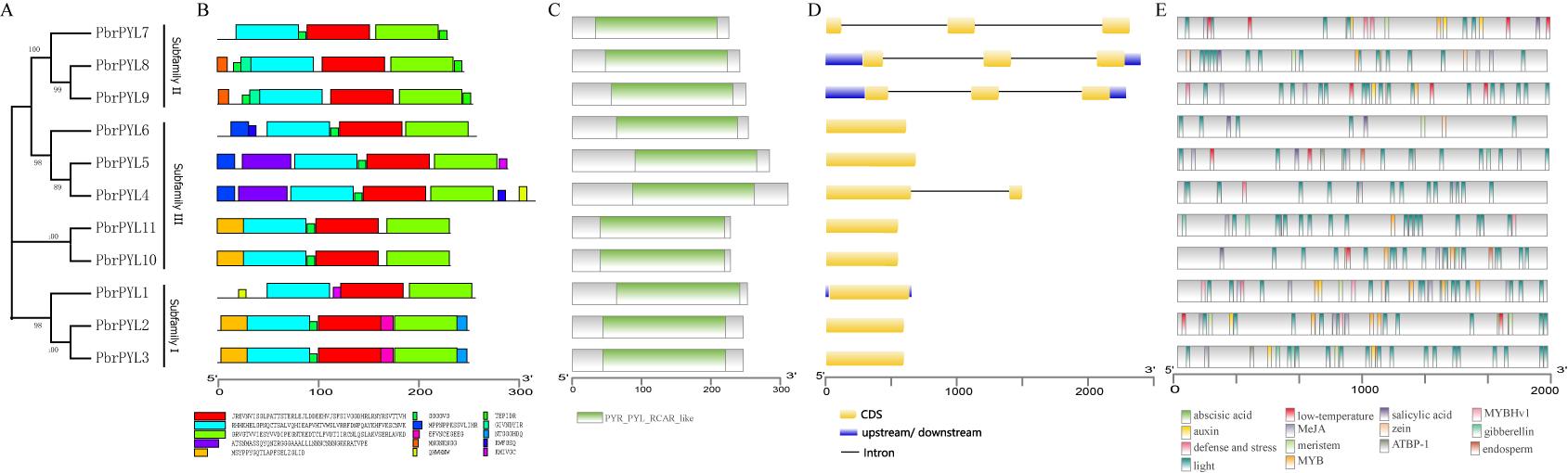


**Supplementary Fig. 1:** Features of *PbrPYL* genes and cis-regulatory elements in the promoter of *PbrPYL* genes.A Phylogenetic relationship. B Distributions of conserved motifs. C Conserved domain. D Exon/intron architectures. E cis-regulatory elements in the promoter of *PbrPYL* genes. Promoter sequences (2000 bp) of *PbrPYL* genes were analyzed by using NewPLACE.
